# The impact of COVID-19 lockdown on postpartum mothers in London, England: An online focus group study

**DOI:** 10.1007/s10389-023-01922-4

**Published:** 2023-05-15

**Authors:** Emily H. Emmott, Astor Gilliland, Anjana Lakshmi Narasimhan, Sarah Myers

**Affiliations:** 1 UCL Anthropology, University College London, 14 Taviton St, London, WC1H 0BW England; 2 UCL Medical School, University College London, London, England; 3 Department of Anthropology and Archaeology, University of Bristol, Bristol, England; 4 BirthRites Lise Meitner Research Group, Max Planck Institute for Evolutionary Anthropology, Leipzig, Germany

**Keywords:** COVID-19, Lockdown, Postpartum, Postnatal, Intensive Mothering, Expert Parenting, Maternal Wellbeing

## Abstract

**Aims:**

This study examines the impact of COVID-19 lockdown on postpartum mothers in England, with the aim of identifying opportunities to improve maternal experience and wellbeing. The postpartum/postnatal period is widely acknowledged as a time when mothers require greater levels of support from multiple sources. However, stay-at-home orders, commonly known as “lockdown,” deployed in some countries to limit COVID-19 transmission reduced access to support. In England, many postpartum mothers navigated household isolation within an intensive mothering and expert parenting culture. Examining the impact of lockdown may reveal strengths and weaknesses in current policy and practice.

**Subject and methods:**

We conducted online focus groups involving 20 mothers living in London, England, with “lockdown babies,” following up on our earlier online survey on social support and maternal wellbeing. We thematically analysed focus group transcripts, and identified key themes around *Lockdown Experience* and *Determinants of Lockdown Experience*.

**Results:**

Participants raised some positives of lockdown, including *fostering connections* and *protection from external expectations*, but also raised many negatives, including *social isolation*, *institutional abandonment,* and *intense relationships within the household*. Potential reasons behind variations in lockdown experience include *physical environments*, *timing of birth*, and *number of children*. Our findings reflect how current systems may be “trapping” some families into the male-breadwinner/female-caregiver family model, while intensive mothering and expert parenting culture may be increasing maternal stress and undermining responsive mothering.

**Conclusions:**

Facilitating partners to stay at home during the postpartum period (e.g., increasing paternity leave and flexible working) and establishing peer/community support to decentre reliance on professional parenting experts may promote positive postpartum maternal experience and wellbeing.

**Supplementary Information:**

The online version contains supplementary material available at 10.1007/s10389-023-01922-4.

## Introduction

Limiting social interactions has been a widely-used public health measure to tackle the COVID-19 pandemic, including stay-at-home orders commonly known as “lockdown” (Arnot et al. 2020). In England, on the 23^rd^ March 2020, the government announced its first national lockdown where individuals were legally required to stay at home with a few exemptions. Non-essential businesses, childcare facilities, and schools were closed; and all public gatherings of more than two people from different households were banned. In terms of health, social care, and community services, while some successfully transitioned to remote delivery (Murphy et al. 2021), many were reduced or closed despite apparent increases in local need (Morton and Adams 2022). Some local councils (local government bodies), in an effort to discourage social gatherings, began blocking access to public areas such as playgrounds, car parks, and public toilets (Duncan et al. 2020). These restrictions continued for over two months until the 1^st^ June 2020, when the phased lifting of restrictions began. Varying restrictions continued throughout 2020-2021, with two further national lockdowns in November 2020 and between January-March 2021 (Brown and Kirk-Wade 2021). Notably, the first national lockdown in England was the strictest, with no allowances for social contact relating to informal support or caregiving (Brown and Kirk-Wade 2021).

These lockdowns inevitably led to social isolation and a reduced availability of support (Arnot et al. 2020; Chiesa et al. 2021). Social isolation may have been particularly challenging for postpartum mothers (up to 6 months after birth; Romano et al. 2010) who ordinarily require support from multiple sources (Myers and Emmott 2021). The birth of a child is often considered a life-changing event (Babetin 2020), with the postpartum period cross-culturally acknowledged as a time when mothers need extra support (Johnston-Ataata et al. 2018). At this crucial time, mothers who had “lockdown babies” experienced reduced access to family, friends, local services and the wider community. This is likely to have had notable impact on maternal postpartum experiences in England.

In England and elsewhere, policymakers have been criticised for implementing COVID-19 measures without attempting to mitigate, or even consider, the impact on those with unpaid care responsibilities, who are predominantly women (Power 2020). This may stem from the male-breadwinner nuclear family norms which situate women as sole caregivers (Sear 2021), compounded by intensive mothering norms which bring childrearing under individual (maternal) responsibility (Shirani et al. 2012). These norms frame mothering as a high-stakes activity with potential for large and long-lasting impact on child development and wellbeing (Shirani et al. 2012), leading to high reliance on parenting experts for information and guidance for the “best mothering practices” (Lee 2014). Within this cultural context, lack of access to trained professionals for maternal and child health support during lockdown may have amplified maternal worry and anxiety. Indeed, a recent study of first-time mothers in the UK during the COVID-19 pandemic suggests that feelings of maternal responsibility were amplified as mothers grappled with uncertainties and infection risk (Gray and Barnett 2022). Our previous survey of postpartum mothers during lockdown in London, England, showed that 47.5% of participants met the threshold for possible postnatal depression (Myers and Emmott 2021), which is notably higher compared to the pre-pandemic UK prevalence of 11-12% (Carson et al. 2015; Petersen et al. 2018). Comparable results of elevated postnatal depression levels have been reported across several studies (Cameron et al. 2020; Harrison et al. 2021; Spinola et al. 2020), indicating that lockdown measures may have been significantly detrimental to maternal wellbeing and mental health.

At the same time, scholars have pointed to how intensive mothering norms are associated with perceived and actual maternal surveillance, as mothers are expected to comply with “best mothering practices” (Fox et al. 2009; Henderson et al. 2010; Shirani et al. 2012). Further, the expectation to follow expert guidance is argued to steer mothers away from following their “maternal instincts,” undermining maternal confidence in the process (Apple 1995; Shirani et al. 2012). Consequently, it is possible that social isolation stemming from lockdown may have presented postpartum mothers with some benefits, by reducing pressures around performing “good mothering.” This is partially captured in an interview study of UK mothers during lockdown, which identified that some mothers felt lockdown freed them from social pressures and obligations (Jackson et al. 2021). Thus, the impact of lockdown on postpartum maternal experience may be complex.

Our previous study of 162 mothers in London, England, highlighted the variability and complexities of lockdown experience, with participants raising positive as well as negative impacts (Myers and Emmott 2021): analysing open-text survey responses, our participants raised how lockdown led to a burden of constant mothering as they were often left to negotiate childcare alone and without breaks. At the same time, the concentrated time within the household facilitated family bonding. With lockdown essentially a “natural experiment” in social isolation, an in-depth understanding of lockdown experience among postpartum mothers may be particularly important for policy and practice, as it could reveal strengths and weaknesses in the current systems which may be otherwise hidden or under-recognised.

Here we conduct a follow-up exploratory study of mothers with “lockdown babies,” drawing on a sub-sample of London mothers from our original survey (Myers and Emmott 2021). By conducting thematic analysis of online focus group data, we explore the experiences of postpartum mothers in London who had and raised babies under extraordinary circumstances. We pay particular attention to similarities and variations in lockdown experiences, including the potential determinants of different maternal experience during England’s first national lockdown. We draw on contemporary mothering culture in England, described as intense, individualised in responsibility and expert-reliant, to interpret our findings; and discuss opportunities for post-lockdown policy and practice to promote postpartum wellbeing.

## Methods

We conducted four online focus groups involving 20 mothers in April 2021, capturing retrospective reflections of their lockdown experiences, and thematically analysed the transcripts. Our preregistered research plan is available at 10.17605/OSF.IO/FB7HQ, outlining our original research questions, study design, sampling strategy, data collection methods, topic guide, analysis process, and credibility strategy. Further information on our applied methods including deviation from the registered research plan, recruitment text, and notable information in relation to data collection is included in the supplementary information. This project was reviewed and approved by UCL Research Ethics (ref: 14733/002), and all participants gave consent to take part.

### Recruitment

Participants were recruited from our pre-existing pool of mothers who took part in our earlier survey on social support during lockdown in London. Mothers were initially recruited to our study if they had an infant aged ≤6m which they gave birth to in London, and still lived in London at the time of the survey (May-June 2020, towards the end of the first national lockdown; n=163). In total, we contacted 63 survey participants by e-mail, of which 22 signed up to take part, with 20 taking part in the online focus groups in April 2021. Mothers were offered a £20 Amazon voucher as a token of thanks. The mean age of focus group participants was 35.65 years, 11 participants (55%) had one child, and 16 (80%) self-reported their ethnicity as White (Table 1) – broadly reflecting our survey participant characteristics. All focus group participants indicated they were cohabiting with a partner at the time of the focus group.

**Table 1:** Descriptive statistics of study participants. The self-reported ethnicity options in our survey were based on the major ethnic group categorisations used by the government in England and Wales, which were the following: White; South Asian/Asian British; Black/African/Carribean/Black British; East Asian/East Asian British; Mixed; and Other – please specify

Mean Age in Years (SD)	35.65 (3.59)	
Minimum Age in Years	28	
Maximum Age in Years	42	
	n	%
Number of Children		
1	11	55
2	7	35
3+	2	10
Ethnicity*		
White	16	80
South Asian/South Asian British	2	10
Black/African/Caribbean/Black British	1	5
East Asian/East Asian British	1	5
Household Income		
£35-45k	3	15
£45-75k	4	20
£75-100k	4	20
Over £100k	9	45

### Data collection

We conducted online focus groups using Zoom, with 4-6 participants per group. The composition of each focus group was based on the availability of participants at specific times and dates. Each focus group lasted roughly 60 mins, with Emmott as the main facilitator and Myers mainly taking notes and occasionally posing follow-up questions. The session began with introducing the project and reaffirming consent, followed by individual introductions and reminder of the different lockdowns that happened over the preceding year. Focus group topics focused on the general experience and consequences of the first lockdown, with follow up questions on social support, bonding, and infant feeding (though, note, bonding and infant feeding received considerably less attention than social support in all sessions). The focus group session plan which served as a topic guide is available in the supplementary information.

Overall, we tried to foster an informal, friendly atmosphere within the focus group, which led to relatively natural and free-flowing conversations. While most of the participants directed their conversation to the researchers (with other participants as the audience), participants occasionally addressed and spoke to other participants directly. Turn-taking between participants occurred throughout, and referring to each other’s experiences was very common. Most participants were at home, with a few participants taking part outdoors (e.g., on a walk, at the playground). All participants were in a relatively private setting (i.e., no other adults nearby), with or without their baby. No major issues emerged during the focus groups, although on a few occasions we could not hear a participant due to temporary connection issues, which was quickly resolved. All participants opted to have their videos on during the focus group, but only audio was recorded.

### Analysis

Audio from the focus groups were auto-transcribed (verbatim) using MS Teams, then pseudonymised and manually cleaned by referring to the original audio. We conducted inductive thematic analysis of the transcribed focus group data: First, all researchers met to discuss the broader project, aims, and preregistered methods. This was followed by initial independent coding of two focus group transcripts each by Gilliland and Lakshmi Narasimhan, using NVivo. A meeting was then held with all researchers to reflect on and discuss emerging themes across the focus groups (see supplementary information for initial emerging themes). Gilliland and Lakshmi Narasimhan then swapped transcripts, and coded these with reference to emerging themes (meaning each focus group transcript was coded independently by two researchers in effort to ensure reliability). A third meeting with all researchers was held to discuss, identify, and agree on the key themes. These key themes were then reviewed and validated by Emmott by cross-referencing back to the full transcripts, with particular attention to negative cases. This led to reorganisation of some themes and sub-themes. Finally, the results were discussed with a participant-equivalent volunteer to ensure validity (i.e., a London-resident mother who had an infant aged 6m or younger during the first national lockdown), followed by a final review by all researchers.

## Results

Our key themes with are outlined in Table 2. Participants raised some positives of lockdown, including *fostering connections* and *protection from external expectations*, but also raised several negatives, including *social isolation*, *institutional abandonment,* and *intense relationships within the household*. It was apparent that some participants had more positive or more negative experiences during lockdown. Potential reasons behind variations in lockdown experience which emerged during our analysis include *physical environments*, *timing of birth*, and *number of children*. The descriptions of the themes and supporting quotes are presented below. All participant names have been pseudonymised.

**Table 2:** Key themes identified from inductive thematic analysis

	Key Themes
Lockdown Experience	POSITIVES during lockdown:
	Fostering connections
	Protection from external expectations
	NEGATIVES during lockdown:
	Social isolation
	Institutional abandonment
	Intense relationships within the household
Determinants of Lockdown Experience	Physical environment
	Timing of birth
	Number of children

### Positives during lockdown

#### Fostering connections

Unsurprisingly, the first national lockdown led mothers to “stay at home” with their infant and partner for prolonged periods. Many mothers in our focus groups discussed how the first national lockdown, where between-household in-person contact was banned, fostered closeness and bonding between parents and baby. In particular, participants whose partners were furloughed or worked from home reflected on how this enabled their partners to experience parenting more fully. These participants felt that lockdown had given their family an opportunity for fathers to be present throughout their baby’s development, leading to a stronger bond. This was highly valued and cherished by participants, in part due to the unexpected nature of greater partner involvement and the appreciation that this was not “typical.”

*...what's been amazing is the fact that my husband's been able to build such a relationship with [the baby] … him being able to see her grow, because he's been working from home pretty much the whole time has been a really lovely, lovely thing. … [without lockdown] he wouldn't have seen her “first bit of broccoli”, and he wouldn't have seen her, you know, crawling for the first step, the first time… (Amy, Focus Group 1: one 15-month-old at time of focus group; gave birth two months before lockdown)*

*… 'cause he works from home… it has been amazing in a lot of ways like I think he's probably bonded with the baby a lot more than I'd imagine a lot of new dads do. (Lucy, Focus Group 3: one 13-month old at time of focus group; gave birth one month before lockdown)*

For some mothers, the physical distance from others imposed by lockdown was overcome by using technology to connect with friends and other mothers/parents in similar situations – although this was not necessarily “easy” nor an equivalent to face-to-face contact (see *Negatives During Lockdown*). Nonetheless, a sense of shared experience facilitated feelings of connectedness with others. For some participants, WhatsApp (mobile messaging application) was highly utilised to connect with other mothers, who in turn acted as a great source of information and emotional/affirmational support when access to healthcare professionals became challenging (see *Negatives During Lockdown*).

*Whenever there was a medical issue, we probably ask each other in the [WhatsApp] group, like ‘what should I do?’… we would reassure each other – like ‘no, no you’re doing the right thing. Check for this.” And we’d all watch out for each other. (Sunny, Focus Group 2: one 15-month-old at time of focus group; gave birth three months before lockdown)*

*...we've been able to keep that contact with people through the Internet, and I have actually met friends through Zoom, which is bizarre … and you know, [I’ve] never actually seen them [or] been in the same room as some of these people, but have had really hearty conversations with them about being a parent. (Amy, Focus Group 1: one 15-month-old at time of focus group; gave birth two months before lockdown)*

Overall, lockdown unexpectedly allowed some couples to deviate from the male-breadwinner family model, where partners were able to stay at home and increase caregiving and household labour contributions. This seemingly brought benefits for partners as well as mothers, fostering stronger connections with the baby. Outside of the family, some participants were able to utilise digital communication to build relationships with other mothers and parents. These relationships were particularly valued as sources of emotional and affirmational support, and the communal exchange of knowledge and experiences led to a sense of connectedness. For some mothers, worries and anxieties stemming from lack of access to healthcare professionals during lockdown (see *Negatives During Lockdown*) was addressed through support from other mothers.

#### Protection from external expectations

Several mothers discussed how lockdown “protected them” from comments, pressures, and obligations around looking after their baby, allowing them to parent how they wanted to without stress, anxiety, and guilt during the newborn period. Some mothers reflected on being “in tune” with the baby, rather than focusing on what should or should not be done, ultimately leading to better experiences for both mother and baby.

*I just kind of went more with the flow and I feel like that for me has made our bond really tight. I feel like he… feels a bit more secure with me working from my instinct rather than getting myself a bit more anxious if I hadn’t... (Beth, Focus Group 3: one 13-month-old at time of focus group; gave birth one month before lockdown)*

*I think one of the best things for me was that we spend a lot of our days like curled up on the sofa together or in the bed… I just got to breastfeed in peace for hours at a time and cuddle him and things like that and that was really nice, without being interrupted. … I didn't have anyone criticising any points of what I've chosen to do, that I was cuddling him too much or breastfeeding him too much, or not enough or whatever. (Sunny, Focus Group 2: one 15-month-old at time of focus group; gave birth three months before lockdown)*

*… I'm quite grateful for not having to try and get myself out the house to do things with a new baby and just actually do things in our own pace. (Nina, Focus Group 2: one 15-month-old at time of focus group; gave birth three months before lockdown)*

Interestingly, the instinctive mothering practices described by participants mirrors characteristics of responsive parenting (i.e., being sensitive to and promptly responding to infant need) which is associated with better child outcomes (Eshel et al. 2006). Had lockdown not been in place, mothers may have faced greater pressures to meet external expectations, detracting from focusing on and responding to their babies’ individual needs. These participant experiences potentially reveal the costs of intensive mothering and expert parenting culture, where the pressures to follow best parenting practices may undermine responsive parenting practices (Fox et al. 2009; Henderson et al. 2010; Shirani et al. 2012).

### Negatives during lockdown

#### Social isolation

While mothers generally reported strengthened bonds within the household (see *Positives of Lockdown*), this was overshadowed by a strong feeling of social isolation. This sense of isolation spanned multiple domains, including friends, family, and the wider community.

Regarding friends and family, many participants described being unable to establish new connections and strengthen existing ones. While some participants described using digital technology to develop and maintain connections (see *Positives of Lockdown*), for many, digital communication felt superficial and sometimes exhausting as they experienced “Zoom fatigue” (Epstein 2020). The lack of face-to-face contact hindered the ability to develop deep and meaningful relationships. For example, one participant talked about the challenges of establishing new friendships with other parents over WhatsApp. Another talked about how participating in video calls without the practical help to manage two children became a source of stress. Joining video calls was not a low-cost activity; rather, it required effort which was burdensome.

Many mothers also highlighted the importance of face-to-face activities, which were seemingly crucial for their sense of belonging to the local community, as well as their baby’s connectedness to others. Some participants reflected on the lost experiences they would “never get back,” and its impact on maternal and child wellbeing.

*… it sounds like it's a minor thing saying, ‘oh, I didn't get to go on play dates or go to baby groups.’ I think people almost laugh at things like that about mums, like it's seen as quite frivolous and silly ... But I just think it is really important for your mental health and missing out on stuff like that makes a huge difference. (Lucy, Focus Group 3: one 13-month old at time of focus group; gave birth one month before lockdown)*

*What I'm worried about is no one else has ever looked after him on their own, apart from his dad obviously. … I'm worried about more than anything in terms of how it's going to impact him with other people, less so with me. (Rose, Focus Group 3: four-year-old and one-year-old at the time of focus group; gave birth during lockdown)*

While the male-breadwinner and intensive mothering norms in England arguably bring childrearing under individual maternal responsibility (Shirani et al. 2012), our participant experiences evidence how mothers require high levels of social connections and support from multiple sources during the postpartum period. Being unable to access the required support had detrimental impacts on participant experiences and wellbeing, as well as fuelling worries about child development.

#### Institutional abandonment

In addition to social isolation, mothers felt neglected and abandoned by institutions which ordinarily should have considered their needs and provided support. Mothers felt facilities were closed and services withdrawn without considering its impact on postpartum women and families. Forced closures of public toilets and playgrounds by local councils were particularly problematic for our participants.

*Another thing I got really upset about was when the only thing you could do was go for a walk, and the council shut all the public toilets. Just after you’d given birth, it was so restricting. … we pulled into a garage, and I burst into tears in front of somebody saying ‘please can you let me go to the toilet’ and the guys said ‘no, you can’t come in’… I’ve never felt so vulnerable in my life. (Sunny, Focus Group 2: one 15-month-old at time of focus group; gave birth three months before lockdown)*

*…When the playgrounds closed, that was literally like, it was just devastating. And I gave birth [in April], so full into lockdown. So here I am, 9 1/2 months pregnant trying to take my toddler to a field to do something, anything. And… I got told off for sitting down on the grass! (Ada, Focus Group 1: one toddler and one-year-old at time of focus group; gave birth during lockdown)*

Regarding healthcare services specifically, the majority of mothers raised difficulties around accessing advice and support from community and primary healthcare. For some, this led to a strong sense of abandonment as they grappled with a new baby without any “expert advice.” Some participants described how the services they were receiving were suddenly and abruptly removed, amplifying their stress and anxiety.

*...her weight dropped massively when [the baby] was first born, over the first couple of days, so we had a lot of health visitor visits and then [suddenly] none. And that for me felt like we've been dropped off the edge of a cliff. (Amy, Focus Group 1: one 15-month-old at time of focus group; gave birth two months before lockdown)*

Note, some mothers were successful in getting community and primary healthcare support during the first lockdown. In part, this was down to a rapid transition to remote practices such as phone/video/e-mail consultations. However, mothers also described a process of proactively seeking support, including employing opportunistic strategies or paying to access private appointments.

*...we were still able to go to the GP for our jabs… I used that opportunity to ask the questions so [I] must have had like verbal diarrhoea ... I made sure that I wrote down all the questions before I went to [the GP] and they would get the nurse in or a doctor in. (Nina, Focus Group 2: one 15-month-old at time of focus group; gave birth three months before lockdown)*

Being proactive was not always met with success, however, and many felt phone consultations were not enough. One participant recounted how phone consultations led to incorrect diagnosis and medication of a minor skin condition for her baby. Another participant who was particularly isolated during lockdown recounted calling health visitors with concerns, but a postnatal visit was denied as her baby was assessed as having no health issues. She did not feel reassured, and was not signposted to any other support, which ultimately led her to feel abandoned. Several mothers acknowledged how the stretched healthcare services in their area prioritised “high risk” cases and babies who were “failing to thrive.”

Where mothers did access face-to-face healthcare services during lockdown, their experiences seemed to be mixed. For example, one participant shared that being forced to attend medical appointments alone felt “de-humanising,” leading her to “dread her appointments” and “struggled to get through them.” In contrast, one participant described their experience of labour as one of the most positive experiences of lockdown.

*…giving birth was like, um, terrifying, but … it was actually the best part of the whole thing, because, um, I felt like I actually got into a safe place [tears up]. They were so nice. It was just me and my husband, but they were like, don't worry, you're here, it's ok… you can do this… (Ada, Focus Group 1: one toddler and one-year-old at time of focus group; gave birth during lockdown)*

Overall, our participant experiences reflect an expectation of continued support from local/national governments and healthcare services throughout lockdown. However, strict government rules severely restricted access to professional support and information, which caused high levels of stress for many postpartum mothers. While our participants did not blame individual professionals such as healthcare staff, the lack of consideration of mothers and families with young children left our participants feeling betrayed and abandoned by policy and decision makers, leading to a sense of anger and injustice.

*… it just felt like the government or whoever just didn’t care, or it just didn’t pass anyone’s mind to help us. (Beth, Focus Group 3: one 13-month-old at time of focus group; gave birth one month before lockdown)*

*I just don’t think there [was] any planning, not just [for] the postnatal mothers, but in general. There just didn’t seem to be any consideration... There are all these different vulnerable groups [with different needs], and we’re all lumped into the same bucket like somebody else, and then had all sorts of healthcare and support just ripped away. (Daphne, Focus Group 2: three-year-old and one-year-old at time of focus group; gave birth 10 days before lockdown)*

#### Intense relationships within the household

While feelings of isolation and abandonment was pervasive during lockdown, this was juxtaposed by the intensity of relationships within the household. Participants were heavily reliant on household members for social connections and support, and some mothers explicitly commented on how they became “too close” with their baby during lockdown as they “couldn’t escape.” When some mothers understandably struggled to look after their baby/children on their own, they sometimes felt let-down by their partner who was their only source of practical support. This intensity caused tension and sometimes resentment, as relationships became draining without any breathing space.

*… there was a bit of me starting to get somewhat resentful of [the baby], and I think that was more because we spent so much time on our own together. It wasn't because of him, it was just [the lack of] those conversations you have, like when you're at children centre… and you can talk to other parents, and that kind of gives you a string of sanity, that you can find other things than your baby. (Mary, Focus Group 1: one 15-month-old at time of focus group; gave birth three months before lockdown)*

*… I think I got a little bit resentful that [my husband] was able to shut himself away and do a full day's work, speaking to grown-ups… and I'd be like downstairs dealing with a 3 year old having a meltdown, trying to calm a newborn baby, and it just would all get a bit too much. (Rose, Focus Group 2: four-year-old and one-year-old at the time of focus group; gave birth during lockdown)*

As briefly explored under *Social isolation*, this reflects the reality of childrearing in England whereby mothers raising children alone is arguably a near-impossible task. Lockdown forced our participants to be sole caregivers for prolonged periods, with detrimental effects on maternal experience and wellbeing.

### Determinants of lockdown experience

While the positives and negatives of lockdown experiences outlined above were broadly shared across our participants, the intensity by which participants experienced them seemingly varied. We identified three potential explanatory factors behind this variation in our data: *physical environments*, *timing of birth*, and *number of children*.

#### Physical environments

The quality of physical environments seemed highly relevant to how stressful participant experiences were during lockdown. Residing in London, many of our participants had limited living space. Some of our participants struggled with being “trapped” in their cramped housing, and several participants raised how the lack of access to outside space was “horrific,” amplifying the normal stressors around looking after babies.

*… at the time we were in a small 2-bed flat with no outdoor space. So it was pretty horrific because you've got a 2-3 year old who wants to run around and be outside, he loves climbing trees, he's an outdoor child. (Sophie, Focus Group 1: three-year old and 14-month-old at time of focus group; gave birth two months before lockdown)*

*… the boat we lived in had one bedroom which basically fitted a double bed and a cot and nothing else. And then the main room, which had everything in it. And my husband was working full time the whole way through, so he's on Zoom at one end of the room, [the baby] wants to go and bash his computer... I mean like... The whole thing was … completely nuts. Now I really look back on it. How on earth did we survive in that one room? (Mary, Focus Group 1: one 15-month-old at time of focus group; gave birth three months before lockdown)*

Weather also seemed to be a large predictor of maternal experiences during lockdown, moderating access to outdoor activities and open space. Many mothers raised the good weather was the “silver lining” during the first lockdown, which meant they could “escape” the stress by going for walks or having socially distanced outdoor encounters with a friend. For some participants, their mood and outlooks seemed to mirror the weather.

*I guess the weather was really nice and it felt, you know… all this pandemic malarky was gonna be over by June (Poppy, Focus Group 4: 13-month-old at time of focus group; gave birth one month before lockdown)*

#### Timing of birth

The timing of birth in relation to the first lockdown seemed to impact mothers in different ways. Some mothers who gave birth just before or at the beginning of lockdown did not particularly feel socially isolated since they were in the “newborn period” where they would have ordinarily stayed at home regardless of restrictions. For some mothers, the newborn period overlapping with stay-at-home restrictions seemed to amplify the positives of lockdown.

*The baby was just born, so we kind of just, heads down, got on with it… and it was sad because nobody could see the baby, but… it was what it was, and you’re in that newborn bit anyway, and my husband was on furlough. So, the first lock down, that was probably horrendous for many, was actually acceptable for us. (Jolene, Focus Group 2: three-year-old and one-year-old at time of focus group; gave birth as first lockdown just started)*

*Well… my husband was at home so I kind of found a bit of a routine, like [the baby] would have a sleep in the morning. Then I basically would just walk for the afternoon, which was really nice, and he was teeny baby then, so he slept quite a lot. (Poppy, Focus Group 4: 13-month-old at time of focus group; gave birth one month before lockdown)*

However, it is important to note that this was not a universal experience. One participant who gave birth during lockdown described how she had been isolated for the last year as she struggled to make friends. Even as lockdown lifted, she did not feel confident about going to baby classes.

*… I did [antenatal classes] with the hope of finding new friends… and then lockdown happened just at the end of March. So our last [class] got cancelled, they all turn into zoom [classes] and we just lost touch. … There were no classes. There was nothing to do. I was on my own at home, and my family and my in-laws were all really far away. And even though lockdown was lifted in the summer, I continued along the same vein… so I didn't actually get out much and do anything… ‘cause I was still feeling quite fearful. (Shelly, Focus Group 4: one-year-old at time of focus group; gave birth during lockdown)*

Our participant experiences suggest that those who had existing support networks or had time to establish connections before lockdown may have fared well being isolated during the “newborn period,” as they were able to reconnect with others as lockdown eased. In contrast, those who were yet to establish new support networks were unable to do so, leaving them vulnerable to prolonged isolation throughout the pandemic. A reflection on the importance of timing of birth was also raised by one participant:

*I think the difference of a few weeks made like a huge difference for people's experience. … I just had that little bit of extra time [before lockdown], just enough time to meet some mums … so within lockdown we could kind of support each other and just kind of meet up for walks. (Molly, Focus Group 4: 16-month old at time of focus group; gave birth four months before lockdown)*

Similarly, mothers who gave birth before lockdown felt lucky as they had access to the healthcare support they required before restrictions kicked in. Interestingly, this did not necessarily reduce the feelings of institutional abandonment, as mothers were still aware of the support which had “disappeared” with lockdown.

*… my son had a severe tongue tie as well and it was found by one of the community midwives. … It did make me feel really sad when we went into lockdown and I was thinking, if I would have given birth like 11 weeks later, I would have struggled to have fed him - and that made me feel sad that there might be women out there that were going through that. (Sunny, Focus Group 2: one 15-month-old at time of focus group; gave birth three months before lockdown)*

Overall, participant experiences point to a possible “sweet spot” regarding the timing of birth: mothers who gave birth just before lockdown may have been able to establish their support networks beforehand, while allowing them to benefit from the protection from external expectations during the newborn period. For these mothers, lockdown may have served a similar purpose to a lying-in period; a cross-culturally common postpartum practice where mothers are protected from social and household obligations to facilitate recuperation and transition to motherhood (Dennis et al. 2007).

#### Number of children

Across focus groups it was apparent that mothers with multiple children were more likely to experience the negatives of lockdown acutely. In many cases, mothers were left to juggle looking after a newborn *and* a toddler/older child with nowhere to go. Some mothers thought this had significant impact on their ability to look after their newborn, as their attention was drawn to meeting their older child’s needs and demands.

*I was thinking a few days ago, you know, I hardly sit down and spend any time sat on the floor with [the baby], doing things, compared to [my first born]… and I know that's [sniffs] um…normal anyway for a second baby, but I would have normally been taking her when he's at nursery in the mornings... (Sophie, Focus Group 1: three-year old and 14-month-old at time of focus group; gave birth two months before lockdown)*

*[I was] trying to pump milk over breakfast time while trying to feed my toddler. I'd have my breast pump attached… and the guilt I felt because I got breast milk into my oldest for five or six months… the guilt I felt because I wasn't able to give [the youngest] the same thing. There's no question that if my oldest had been in nursery, I would have been able to breastfeed for longer, and I feel terrible about that. (Daphne, Focus Group 2: three-year-old and one-year-old at time of focus group; gave birth 10 days before lockdown)*

Interestingly, even though mothers with multiple children were recounting clearly stressful and distressing experiences of lockdown, they believed first-time mothers would have been in worse situations. In part, this was because mothers with multiple children knew what they had “lost” to lockdown.

*First time round, we'd be going and getting children weighed, and then you'd be able to see a health visitor and say, “Oh, by the way, is this normal? Should I go to my doctor or not?” Whereas this time around there was no contact from health visitors, there was nothing. … no one ever sent an email or a call or a text to say, “Are you ok?” which has been quite difficult. [chokes up] (Sophie, Focus Group 1: three-year old and 14-month-old at time of focus group; gave birth two months before lockdown)*

*I felt so grateful I was a second time mother, so I knew what had been discussed in those first round of meetings and what to lookout for and to check for the way and things like that so, you know, I really feel for those ladies who were first time mothers. (Daphne, Focus Group 2: three-year-old and one-year-old at time of focus group; gave birth 10 days before lockdown)*

This contrasted with the actual experiences of some first-time mothers, with many of them feeling they were not as distressed through lockdown as they did not have previous experiences to compare to. As one first-time mother responded to second-time mothers in the focus group:

*It's interesting listening to [second-time mother participants] ‘cause in a way, I'm protected by my ignorance. I'm not aware of quite how abandoned we have been. (Mary, Focus Group 1: one 15-month-old at time of focus group; gave birth three months before lockdown)*

These participant experiences and reflections likely evidence the impact of expert parenting culture, revealed through the natural experiment of lockdown: second-time mothers who had previously experienced support from healthcare professionals viewed expert guidance as crucial for good mothering and child wellbeing. Reassurances from professionals were viewed as important in addressing low maternal confidence and anxiety, and the lack of access to professionals during lockdown may have amplified their stress. In contrast, first-time mothers who did not have similar expectations regarding expert guidance were not negatively impacted by its absence. This is in line with what has been previously argued regarding expert parenting culture; that it may increase maternal anxiety and undermine maternal confidence (Apple 1995; Lee 2014; Shirani et al. 2012).

## Discussion

Overall, our findings point to complex experiences of the first national lockdown within our sample of mothers in London, both positive and negative, with several potential factors relating to variations in experience. Positive impacts of lockdown included *fostering connections* and *protection from external expectations*, while the negative impacts of lockdown included *social isolation*, *institutional abandonment,* and *intense relationships within the household*. Negative impacts were more likely to have been amplified when participants experienced poor physical environments, for example due to cramped housing situations and lack of access to outside space, and when participants had multiple children to look after. Positive impacts were more likely to have been amplified when participants experienced lockdown during the newborn period, contingent on participants having previously established a support network.

The unprecedented and sudden social isolation experienced by postpartum mothers in London was clearly very stressful for many. Nonetheless, lockdown essentially serves as a “natural experiment,” pressure-testing the current systems around postpartum mothers to help identify strengths and weaknesses. Understanding the positives and negatives of lockdown may therefore reveal opportunities for future policy and practice which may allow us to promote maternal experience and wellbeing more effectively.

First, our results *(Fostering connections)* show that lockdown allowed some partners to spend more time at home, which serendipitously increased their contribution to parenting and childcare. Similar findings from Italy have also been reported (Mangiavacchi et al. 2021). This suggests that, outside of lockdown, fathers may be willing and wanting to provide higher levels of childcare; yet, they are unable to do so due to conflicts with work, “trapping” families into the male-breadwinner/female-caregiver family model. Indeed, the conflicts between parenting, domestic labour, and paid employment for both men and women are well-evidenced (Molina 2021). Our results are therefore in line with previous studies which suggest that facilitating fathers to stay at home through flexible/home working and longer paid paternity leave may reduce work-family conflict, lessen the burden of childcare and household labour for mothers, while helping to foster family connectedness (e.g., see Petts et al. 2020; Tanaka and Waldfogel 2007; Molina 2021).

Second, our results reveal the consequences of intensive mothering and expert parenting culture in England. As described in *Protection from external expectations*, lower levels of perceived surveillance during lockdown seemingly allowed freedoms in maternal practice among our participants, which in turn facilitated responsive parenting as participants focused on meeting their baby’s individual needs rather than “what they should be doing.” Responsive parenting has been associated with better socio-emotional, behavioural, cognitive and health outcomes in children, and encouraging responsive parenting has been suggested as a cost-effective means of improving public health (Eshel et al. 2006). As an example, in the UK, responsive feeding is promoted as an optimal feeding practice, and mothers are encouraged to be sensitive to infant feeding cues and to respond to them promptly (UNICEF UK 2016; Black and Aboud 2011). However, our results point to a somewhat ironic conflict whereby public health promotion of responsive mothering practices by professional healthcare experts may undermine instinctive (and naturally responsive) mothering practices: when experts point to the “correct” ways to care for infants, maternal attention may be turned to following the guidance to perform good mothering, detracting focus from infants (Apple 1995; Shirani et al. 2012; Lee 2014). This may explain, for example, why breastfeeding rates in England remain one of the lowest in the world despite decades of public health promotions (Emmott 2023): breastfeeding is more likely to be successful where mothers feed to meet the varying needs of infants (UNICEF UK 2016, Black and Aboud 2011), yet reliance on expert information from healthcare professionals may inadvertently signal that there is a “correct method” to feed, undermining maternal instincts and confidence (Emmott 2023).

The consequences of intensive motherhood and expert parenting norms are further revealed through postpartum maternal experiences surrounding access to healthcare services (*Institutional abandonment)*. The national lockdown forced many services to focus on “high risk” cases (Morton and Adams 2022), introducing challenges for mothers in accessing information and reassurances around their baby’s general health and development, which in turn left mothers to feel “abandoned by the system.” Again, these results may reflect the reliance of contemporary Western mothering on expert advice (Shirani et al. 2012; Lee 2014). However, our results *(Number of children)* also suggested that the stress and anxiety around lack of access to expert healthcare professionals was greater for second-time mothers who had previously experienced, thus expected, such support. First-time mothers who had not expected such support were not necessarily distressed by lack of routine access to healthcare professionals.

While our results highlight the perceived importance of healthcare practitioners as parenting experts in England, our results also raise the question of whether this is something we should continue to promote in an over-stretched healthcare system, particularly as the culture of relying on parenting experts may devalue maternal instincts and lower maternal confidence (Shirani et al. 2012; Lee 2014). This is not to say that healthcare practitioners do not provide adequate support and information to postpartum mothers, or that professional support is never needed. Rather, for *general* postpartum support, framing healthcare professionals as parenting experts and directing mothers to seek their support for any issue may not be the most effective pathway to improve maternal experience and wellbeing. Instead, we hypothesise that encouraging peer and community support networks may counter the culture of expert parenting while ensuring support is readily accessible.

Investing in peer and community support also aligns with our findings identifying the importance of support from beyond the nuclear family, including family, friends, and the wider community (*Social isolation; Intense relationships within the household)*. Social isolation and lack of support has been associated with postnatal depression (Mauthner 1995; Myers and Emmott 2021; Taylor et al. 2022), with 47.5% of participants in our original survey meeting the threshold for postnatal depression (Myers and Emmott 2021). A recent study suggests that diversity in social interactions predicts subjective wellbeing, over-and-beyond social network size (Collins et al. 2022). Facilitating a balanced support system around mothers, and decentring parenting experts as primary sources of support, may enhance postpartum maternal experience and wellbeing.

Finally, our study points to variations in postpartum lockdown experience depending on *Physical environments*, *Timing of birth*, and *Number of children*. London mothers with greater access to indoor and outdoor space were better able to “escape” the stressors of lockdown, which point to the potential importance of access to open areas and green space for mental wellbeing (Wendelboe-Nelson et al. 2019). Further, mothers who experienced lockdown during the “newborn period” seemed more likely to experience the benefits of lockdown. The immediate postpartum period is commonly recognised as a time when mothers require rest, reflected by lying-in rituals and customs across cultures (Dennis et al. 2007; Johnston-Ataata et al. 2018). While such customs are less prevalent in Western societies, our results point to the benefits they may have for mothers, including protection from social obligations and parenting pressures. Most notably, our results suggest that mothers with multiple children experienced higher degrees of stress than first-time mothers during lockdown. At the same time, these mothers perceived first-time mothers to have had a worse experience – perhaps reflecting the assumed vulnerability of first-time mothers. In our previous pre-pandemic study, mothers with multiple children were less likely to receive high levels of support from across family, friends, and health professionals (Emmott et al. 2020) despite arguably “having more to deal with.”

## Limitations

This study builds on our previous analyses of survey data which focused on social support during lockdown and maternal mental health (Myers and Emmott 2021). Consequently, our participants were initially informed that our study focused on social support and maternal wellbeing, and our data and study findings are likely to be biased towards themes relating to these topics. At the same time, while some participants raised issues around their mental health, we were unable to directly pose questions on this topic due to restrictions stemming from our ethics-approval. Overall, this means our focus group data around maternal mental health and wellbeing during lockdown may not be comprehensive.

Further, our focus groups occurred just over a year after the first lockdown was announced, meaning all accounts are retrospective and prone to bias. We note that experiencing two further lockdowns over the course of a year provided comparison points, which seemed to help mothers process and explain the issues and challenges of the first lockdown which was particularly strict. Crucially, our study is limited to London, which was the epicentre of the initial COVID-19 spread in England - which likely resulted in different levels of perceived risk and responses compared to other areas. Our participants were mostly White, and 45% of our participants self-reported a household income of over £100,000 (equating to approximately £70,000-80,000 equivalised household income), placing them in the top 20% of household incomes in the UK (based on 2021 data; Office for National Statistics 2023). While our findings are therefore not generalisable; we hope they serve as a useful case study on the impact of social isolation on postpartum mothers. Finally, it is important to note that our sample of mothers were all cohabiting with their partner at the time of data collection. It is unlikely that our results fully reflect the experiences of mothers across diverse household structures, such as single-parent and three-generational households.

## Conclusion

Our study evidences the experiences of the first national lockdown among postpartum mothers in London, England, under the culture of intensive mothering and expert parenting. It highlights the high costs of lockdown endured by many mothers, leading to a sense of abandonment by policymakers and service providers. At the same time, our study reveals some positives of lockdown, including a greater sense of connectedness within the household when partners were able to stay at home, as well as protection from external expectations and maternal surveillance. In light of our findings, policy and decision makers should consider better promotion of peer and community support networks for postpartum mothers, decentring expert advice for general maternal practice. Additionally, offering greater flexibility in work arrangements and increasing paid paternity leave could help fathers play a more active role in parenting, improving family relationships and postpartum maternal wellbeing.

## Supplementary information

ESM 1 (DOCX 60.3 KB)

## Data Availability

Our data is not publicly available due to risk of participant identification and limits to consented data usage. Data may be shared with researchers upon reasonable request; please contact the corresponding author for more detail.
