# Supplementary material for: The impact of COVID-19 lockdown on postpartum mothers in London, England: An online focus group study
Source: Z Gesundh Wiss. 2023 May 15:1–13. Online ahead of print. doi: 10.1007/s10389-023-01922-4 (PMC10184075; doi:10.1007/s10389-023-01922-4)
Supplement: Supplementary file 1 — (DOCX 60.3 KB) [file 10389_2023_1922_MOESM1_ESM.docx]

Coping with ‘Lockdown Babies’: Understanding Postpartum Maternal Experience in London, UK

Supplementary Information

Contents

[Preregistration Information 2](#_Toc119485022)

[Deviation from registered methods 2](#_Toc119485023)

[Research questions 2](#_Toc119485024)

[Recruitment 2](#_Toc119485025)

[Data analysis process & credibility strategies 2](#_Toc119485026)

[Further information on recruitment 3](#_Toc119485027)

[Further information on data collection 4](#_Toc119485028)

[Further information on data analysis 5](#_Toc119485029)

[Thematic analysis 5](#_Toc119485030)

[Emerging Themes 5](#_Toc119485031)

# Preregistration Information

The preregistered data collection and analysis methods, including the topic guide, are available at [OSF Registries | Social Support and Maternal Wellbeing through the National Lockdowns in England](https://osf.io/fb7hq). Note, the registration date is listed as 27^th^ January 2022 as Emmott missed pressing the final registration button. However, the original preregistration document was last amended on the 29^th^ March 2021.

# Deviation from registered methods

### Research questions

We decided to disregard our fourth research question, “How did the different lockdowns and the support impact infant feeding?,” as we felt there was not enough data in the focus group to fully explore this question.

### Recruitment

1. Our pre-registration document stated we had originally recruited 162 survey participants. This was incorrect; we had originally recruited 163, but one participant did not complete the full survey. This did not impact the final eligible sample for the focus group, which was 136.
2. Our original recruitment strategy was to conduct purposive sampling based on self-reported experience of support and infant bonding, aiming to recruit an equal number of mothers who reported: 1) strong infant bond and low support, 2) strong infant bond and high support, 3) weak infant bond and low support, and 4) weak infant bond and high support. Post-recruitment and analysis, Myers identified an error with the participant survey responses used for focus group recruitment, which meant that the coding for support and infant bonding did not match the original participant survey responses. In essence, this means our participant recruitment was random rather than purposive.

### Data analysis process & credibility strategies

1. Two additional researchers Gilliland and Lakshmi Narasimhan joined the project, and were fully briefed about the project and methods. They led on the inductive thematic analysis with support from Myers and Emmott.
2. While our initial plan stated that we would not fully transcribe the focus groups, this planned method became more challenging to coordinate due to more researchers joining the project, and transcription became feasible due to auto-transcription features being made available on MS Stream (which was available to us via our institution). The audio files were therefore initially auto-transcribed securely using MS Stream. The audio files were then re-listened to by Gilliland and Lakshmi Narasimhan, who then fully anonymised and edited the focus group transcripts. These transcripts were then coded and thematically analysed.
3. All researchers discussed preliminary and final findings throughout the analysis process.
4. Due to the large gap between data collection and finalisation of the analysis and time constraints, we did not seek direct feedback from participants to inform our final findings. Instead, our findings were shared with subject-equivalents in our social network to seek their thoughts and ensure credibility in findings.

# Further information on recruitment

*The following text was sent to recruit participants from our original survey who indicated that they would like to take part in a focus group.*

Dear participant,

Last year you took part in our survey on social support and maternal wellbeing during lockdown, and expressed that you would like to take part in a focus group to share your thoughts and experiences. **We are contacting you today as we would like to invite you to take part in a 1hr online focus group on Zoom, which will involve a group discussion with other mothers.**

The online focus groups will happen on [DATES DELETED TO ENSURE CONFIDENTIALITY]. If you would like to take part, please let us know when you will be available by filling out this form by this Sunday (4^th^ April): [ORIGINAL LINK DELETED]

During the focus group, project researchers will ask some questions about your experience during the different lockdowns. The audio from the session will be recorded (not the video) and used for research purposes. Participation is voluntary, and anything you decide to share during the focus group will be anonymised by the researchers (meaning we won’t identify who you are in any of our publications). You will receive a £20 Amazon voucher after the focus group as a token of thanks.

**So you can decide if you want to take part, here is some additional important information about the focus group you should know:**

**What is the research about?**

It’s been just over a year since the first national lockdown, and we would like to know about your experiences over the different lockdowns. Unlike in the survey, we won’t be asking you specific questions about your mental health, though you are free to talk about this if you wish.

**Why have we contacted you?**

We are contacting different mothers to get a variety of views and experiences, including different levels of support, wellbeing, household income and ethnicity.

**What will happen in the online focus group?**

You’ll be in a group with 3-5 other mothers who also took part in the survey. We hope the discussion will be friendly and informal, and you are free to say as little or as much as you want. The focus group will take about an hour of your time, and we will be recording the audio of the conversation (but not the video). The recording will only be used for research purposes, and it will not be shared with anyone outside this study.

As privacy measures, the Zoom meeting will be password protected, and we will use the waiting room function to manually admit people into the conversation.

**Do I have to take part?**

No, you don’t have to take part if you don’t want to. Even if you decide you want to take part, you can decide not to participate at any time, including during the focus group.

**How will you protect my data?**

We will not be able to guarantee complete confidentiality, because we can’t prevent other participants talking about what was discussed. However, anything you decide to share during the focus group will anonymised by project researchers, meaning we won’t identify who you are in any of our publications.

This study is GDPR compliant, and UCL’s general privacy notice for participants in health and care research studies can be [found here](https://www.ucl.ac.uk/legal-services/privacy/ucl-general-privacy-notice-participants-and-researchers-health-and-care-research-studies). For data protection enquiries please contact UCL’s Data Protection Officer Alex Potts on [e-mail redacted].

**Are there benefits for taking part?**

If you decide to take part and join the focus group, you will receive a £20 Amazon voucher as a token of thanks. You will receive this regardless of how much you take part in the focus group. For example, if you decide you don’t want to say anything, or decide that you don’t want to participant half-way through, you will still receive the voucher.

**Who has approved this project?**

This project has been approved by the UCL Research Ethics Committee (ref:14733/002). If anything goes wrong, or you want to complain, you can contact them at [ethics@ucl.ac.uk](mailto:ethics@ucl.ac.uk). If you would like more information about the study, you can contact Dr Emily Emmott or Dr Sarah Myers at [e-mail address no longer available].

**How do I sign up?**

The online focus groups will happen on Friday 9th April and Saturday 10th April. If you would like to take part, please let us know when you will be available by filling out this form by this Sunday (4th April): [link no longer available]

Best wishes,

Dr Sarah Myers and Dr Emily Emmott

# Further information on data collection

Mothers were initially recruited to our study if they had an infant aged 6m or younger which they gave birth to in London, and still lived in London at the time of the survey (May-June 2020) (n=163). Of these women, 136 said they would be willing to take part in a focus group and provided complete survey responses on social support, infant bonding, income, age, number of children and ethnicity.

# Focus Group Session Plan

## Part 1: Introduction/background, consent, ground rules

- Hi everyone, thank you so much…
- Just to **introduce myself** briefly, I’m Emily and colleague Sarah, we are the faces behind the survey you have kindly taken part in.
- Before we start… **overview of what’s happening today…** and please ask questions if you have any:
  - As you know… study on social support and maternal experience during lockdown. Today I’m hoping we can have a group discussion around your experiences over the last year to get a more detailed understanding.
  - I will mainly be asking the questions, and Sarah might follow up on some things.
  - Just want to say that this it **totally voluntary** – so if you’re not feeling it that’s fine, please don’t feel obliged to share things you don’t want to. You are welcome to turn your camera off if you need a little break – do what makes you comfortable. And when we write up our research everything will be anonymised, so we won’t identify you in any of our published material.
  - Another thing to keep in mind is that **people’s experiences are often different**, and there are **no right or wrong answers** to any of the topics or questions – so I am hoping this would be **a friendly session** where we listen to each other and see what we all have to say.
  - **And in terms of speaking**, we’re quite a small group so hopefully we can just have a chat like normal – but if you’ve got something you want to say, feel free to use the raise hand button. And if there’s a lot of feedback I might have to mute some people, so don’t forget to unmute when you are speaking.
  - Before we start, just to remind you that we are going to record the audio of the session, but not your lovely faces. So I might say your name quite a lot so we know who is talking, which might feel a bit weird, but let’s see how it goes.
- Does that sound ok? Do you have any questions?

****Start recording here****

- Icebreaker: Before we start, I thought we should get to know each other – introduce yourselves. (5 mins)
  - I will go first (Emily: introduce where you live, who you live with; pass on to participants, end with Sarah])
- Explain Lockdown 1, Lockdown 2, and Lockdown 3 (2 mins)
  - Lockdown 1: 23rd March-28th May 2020
  - Lockdown 2: 5th November - 2nd December 2020
  - Lockdown 3: 20^th^ December 2020-29^th^ March 2021

## Part 2: Lockdown Experience

1. To start off, I was wondering if you could share what your experience was like during the different lockdowns? [General Q. Ensure researcher ask about participant household situation for context]

[follow up with social support questions]

- 1. How did you keep in contact with people? [**Did you contact them; did they contact you**]
  2. What kind of support did you find really helpful?
  3. What didn’t you find very helpful / what could have been better?
  4. Did things change once lockdown was eased?

1. Overall, how did the different lockdowns impact you and/or your family? (10 mins)

[follow up with bonding questions]

- 1. How do you think lockdown impacted your bond with your baby? [Ensure researcher follows up with “why,” if required]

[follow up with infant feeding questions]

- 1. I just had one last question – lots of mums have different experiences of feeding babies, and I wanted to hear your thoughts on if lockdown impacted anything around feeding your baby? [Ensure researcher ask about intention to breastfeed and mode of feeding if not clear] (10 mins)

1. Would anyone like to share anything else about being a mum with a baby during COVID? (5 mins)

*****Stop recording here*****

## Part 3: Wrap-up

Thank participants, explain next steps/what will happen. Any questions? (3 mins)

- Thank you so much for your time
- So we are running a few more focus groups with other mums, and once that is finished we will be analysing the data -which is a long process.
- If you want to keep updated the easiest is probably to follow us on twitter because we share our study findings through that, but you can always contact us and we are happy to give an update
- And we will e-mail you your £20 amazon voucher by the end of today.
- Any questions?

# Further information on data analysis: Emerging themes

The following table outlines the emerging, preliminary themes which stemmed from the first group meeting where all researchers reflected on and discussed the initial coding. These themes were revised, reorganised and condensed into the final themes as outlined in our results section.

|  | **Main Theme** | **Sub Theme** |
| --- | --- | --- |
| **Buffer (Benefits / Pros)** |  |  |
|  | Feeling more connected to baby / bonding / secure attachment |  |
|  | Partners (fathers/husbands) spending more time with baby and children |  |
|  | Lack of judgement / lack of pressure | Infant feeding |
|  | “High risk/severity” emergency/acute Healthcare |  |
| **Stressor (Costs / Cons)** |  |  |
|  | Lack of support | Loneliness / Isolation / lack of connectedness |
|  |  | “Low risk/severity” lack of healthcare |
|  | Disruptions to plans / uncertainty | Disappointment from change of plans (e.g., maternity leave) |
|  |  | Not being able to make plans |
|  | Need for self-reliance & self-advocacy |  |
|  | Chaos / Armageddon |  |
|  | Lack of space | Nowhere to go |
| **Outcome** |  |  |
|  | Mismatch expectation vs reality | Infant feeding |
|  | Worries about baby and attachment | Too attached to mum |
|  |  | Not attached to others |
|  | Maternal mental health & wellbeing | Overwhelmed |
|  |  | Exhaustion |
|  |  | Disappointment |
|  |  | Guilt |
|  |  | Gratitude |
|  |  | Maternal Empowerment |
| **Variations in experience (case studies?)** |  |  |
|  | Timing of birth/ first baby? |  |
|  | Parity |  |
|  | Finding new connections? |  |
|  | Healthcare support |  |
